# X-linked Charcot-Marie-Tooth disease, Arts syndrome, and prelingual non-syndromic deafness form a disease continuum: evidence from a family with a novel *PRPS1* mutation

**DOI:** 10.1186/1750-1172-9-24

**Published:** 2014-02-14

**Authors:** Matthis Synofzik, Jennifer Müller vom Hagen, Tobias B Haack, Christian Wilhelm, Tobias Lindig, Stefanie Beck-Wödl, Sander B Nabuurs, André BP van Kuilenburg, Arjan PM de Brouwer, Ludger Schöls

**Affiliations:** 1 Department of Neurodegenerative Diseases, Hertie-Institute for Clinical Brain Research, University of Tübingen, Tübingen, Germany; 2 German Research Center for Neurodegenerative Diseases (DZNE), Tübingen, Germany; 3 Institute of Human Genetics, Technische Universität München, Munich, Germany; 4 CeGaT GmbH, Center for Genomics and Transcriptomics, Tübingen, Germany; 5 Diagnostic and Interventional Neuroradiology, Department of Radiology, University Hospital Tübingen, Tübingen, Germany; 6 Institute of Medical Genetics and Applied Genomics, University of Tübingen, Tübingen, Germany; 7 Computational Drug Discovery group, Center for Molecular and Biomolecular Informatics, Radboud University Medical Centre, Nijmegen, The Netherlands; 8 Genetic Metabolic Diseases, Academic Medical Center Laboratory, Amsterdam, The Netherlands; 9 Department of Human Genetics, Donders Institute for Brain, Cognition and Behavior, Radboud University Medical Centre, Nijmegen, The Netherlands

**Keywords:** Ataxia, Early onset ataxia, Genetics, Hearing loss, Optic atrophy, Behr syndrome

## Abstract

**Background:**

X-linked Charcot-Marie-Tooth disease type 5 (CMTX5), Arts syndrome, and non-syndromic sensorineural deafness (DFN2) are allelic syndromes, caused by reduced activity of phosphoribosylpyrophosphate synthetase 1 (PRS-I) due to loss-of-function mutations in *PRPS1*. As only few families have been described, knowledge about the relation between these syndromes, the phenotypic spectrum in patients and female carriers, and the relation to underlying PRS-I activity is limited.

**Methods:**

We investigated a family with a novel *PRPS1* mutation (c.830A > C, p.Gln277Pro) by extensive phenotyping, MRI, and genetic and enzymatic tests.

**Results:**

The male index subject presented with an overlap of CMTX5 and Arts syndrome features, whereas his sister presented with prelingual DFN2. Both showed mild parietal and cerebellar atrophy on MRI. Enzymatically, PRS-I activity was undetectable in the index subject, reduced in his less affected sister, and normal in his unaffected mother.

**Conclusions:**

Our findings demonstrate that CMTX5, Arts syndrome and DFN2 are phenotypic clusters on an intrafamilial continuum, including overlapping phenotypes even within individuals. The respective phenotypic presentation seems to be determined by the exact *PRPS1* mutation and the residual enzyme activity, the latter being largely influenced by the degree of skewed X-inactivation. Finally, our findings show that brain atrophy might be more common in *PRPS1*-disorders than previously thought.

## Background

Charcot-Marie-Tooth disease-5 (CMTX5, MIM 311070 [1,2]), Arts syndrome (MIM 301835 [3,4]) and X-linked nonsyndromic sensorineural deafness (DFN2; MIM 304500 [5]) present three clinically distinct but genetically allelic disorders, caused by reduced phosphoribosylpyrophosphate synthetase 1 (PRS1) activity due to *PRPS1* mutations [6]. Only three families with CMTX5 and two families Arts syndrome, respectively, have been reported worldwide so far [1,3,7]. Thus, evidence is still rare whether these two disorders are separate entities, or rather clusters on a phenotypic continuum of *PRPS1*-related disease. In addition, knowledge about intrafamilial variability and phenotypic manifestations in female carriers is limited.

Here, we report a family with a novel *PRPS1* missense mutation providing several new insights in PRS-I -hypoactivity disease. First, features of CMTX5 and Arts syndrome features can overlap within individuals, indicating an intraindividual continuum of these two disorders. Second, CMTX5/Arts and (prelingual, severe) DFN2 can present within one and the same family, revealing an intrafamilial continuum of these disorders. Third, the respective phenotypic presentation along the continuous spectrum of *PRPS1*-related disease seems to be determined by the exact *PRPS1* mutation and the degree of residual PRS-I enzyme activity, the latter being largely influenced by the degree of skewed X-inactivation.

## Methods

The index patient, his sister and his mother were investigated by (i) detailed clinical examination by a specialist in rare neurogenetic disorders, (ii) routine laboratory serum tests including uric acid analysis, (iii) enzymatic analyses of PRS-I activity in erythrocytes (for methodological details, see Additional file 1), and (iv) *PRPS1* DNA sequence analysis (for methodological details, see Additional file 2). Additionally, the index patient and his sister were assessed by (v) nerve conduction studies, (vi) ophthalmological funduscopy, and (vii) routine brain magnetic resonance imaging (MRI).

The frequency of the identified *PRPS1* variant was determined in more than 4,250 X-chromosomes of European control individuals with unrelated phenotypes analyzed by exome sequencing (for methodological details, see [8]). In addition, the frequency of the change was assessed in the exome variant server (EVS) of the National Heart, Lung, and Blood Institute GO Exome Sequencing Project (Seattle, WA, USA; URL: evs.gs.washington.edu/EVS/) with the corresponding nucleotide positions being analyzed in >8,000 European and >4,000 African American alleles, and in the Database of Single Nucleotide Polymorphisms (dbSNP; Bethesda: National Center for Biotechnology Information, National Library of Medicine [dbSNP Build ID: 138]; available from: http://www.ncbi.nlm.nih.gov/SNP/). *In-silico* predictions of the pathogenicity of genetic variants were performed using PolyPhen-2 [9], SIFT [10] and MutationTaster [11]. The effect of the amino acid change on the structure of *PRPS1* was predicted using the crystal structure of human *PRPS1* from [12] (PDB entry 2H06; http://www.pdb.org/) as described before [4]. X-inactivation patterns in female *PRPS1* mutation carriers were tested by using the highly polymorphic (CAG)n region of the human androgen receptor gene (HUMARA locus) after methylation-sensitive digestion with HpaII, as described previously [13]. This study was carried out in compliance with the Helsinki Declaration, and approved by the Institutional Review Board of the University of Tübingen, reference number 598/20118O1.

### Consent

Written informed consent was obtained from the patients for the publication of this report and any accompanying images.

## Results

### Clinical assessment

**Figure 1:**
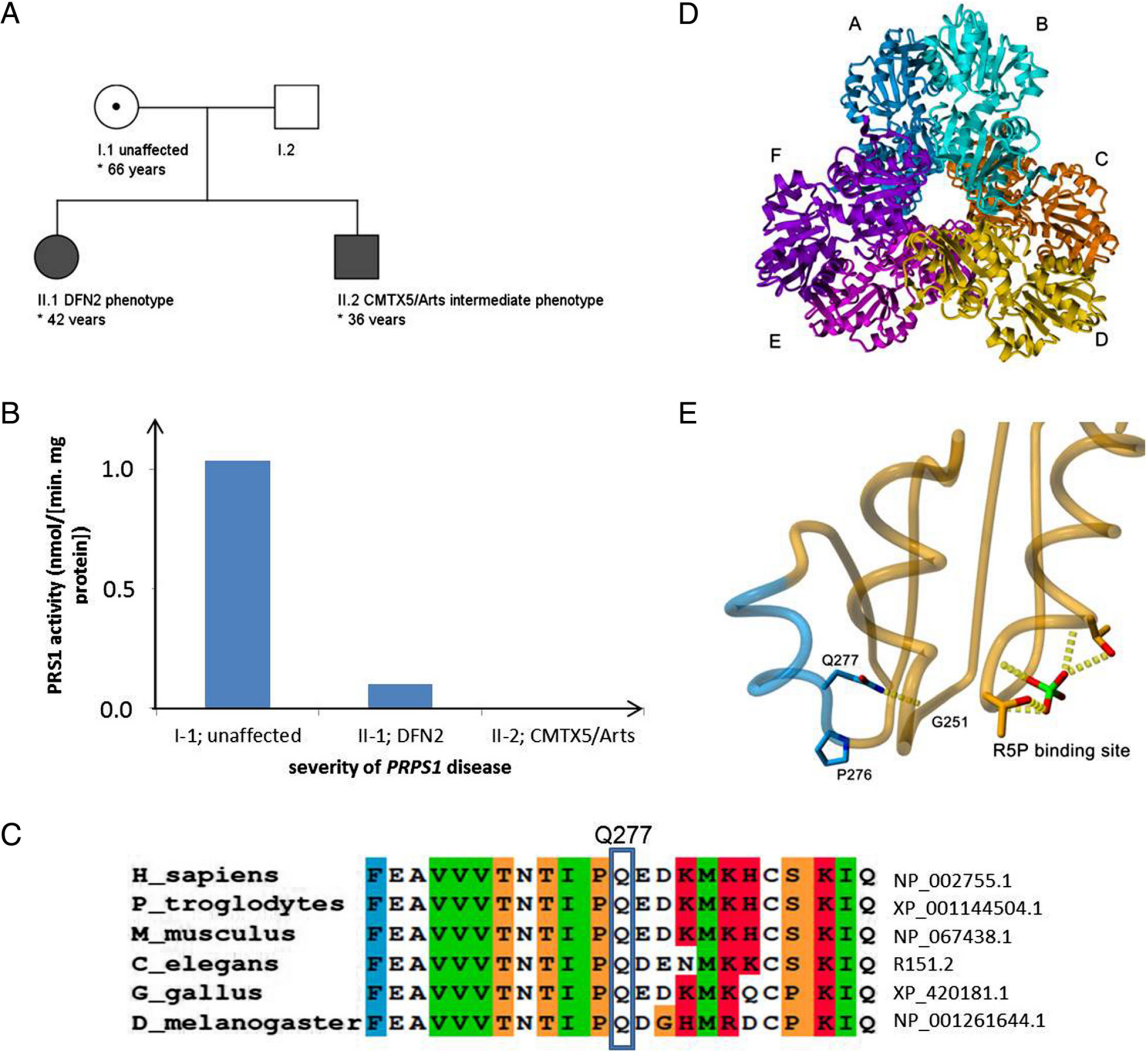
**Pedigree, PRS enzymatic activity, conservation, and predicted protein structural effects of the novel Gln277Pro *PRPS1 *variant. (A)** Pedigree of the family shows maternal inheritance of the *PRPS1* mutation with variable phenotypic severity of disease, reaching from an unaffected state in the mother (I-1) to a mildly affected DFN2 phenotype in the sister of the index patient (II-1) to a severe Arts/CMTX5 overlap syndrome in the index patient (II-2). **(B)** The severity of the phenotype correlated with the PRS1 enzymatic activity in erythrocytes. **(C)** Protein alignment demonstrates high conservation for the mutated amino acid position 277 in the PRPS1 protein across mammals and non-mammals. **(D)** PRS1 catalyses the synthesis of phosphoribosylpyrophosphate (PRPP) from ribose 5-phosphate (R5P) and ATP, and is thought to be physiologically functional as a hexamer, consisting of three homodimers arranged in a propeller-like shape. In this hexamer, the N-terminal domains are located at the centre and the C-terminal domains at the outside. **(E)** Gln277 is predicted to interact with Gly251, which is close proximity to the R5P binding site The p.Gln277Pro change will result in a loss of this hydrogen bonding interaction potentially destabilizing the region surrounding the R5P binding site, thus affecting the *PRPS1* catalytic active site.

**Figure 2:**
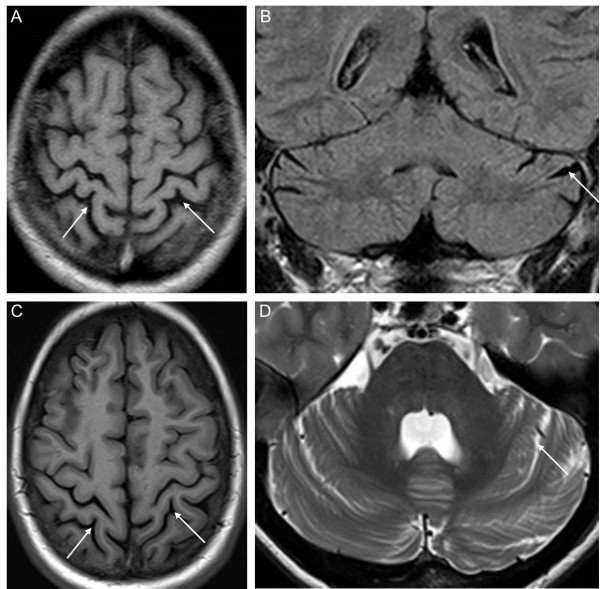
**Brain magnetic resonance imaging (MRI) of the two affected siblings.** MRI shows diffuse parietal atrophy (arrows in **A**, **C**) and mild cerebellar atrophy (arrows in **B**, **D**) in the index patient II-2 at age 31 years (upper row) as well as in his sister II-1 at age 38 years (lower row) (**A**, **C** T1 axial; **B**, FLAIR coronar; **D** T2 axial).

A 36 year-old male patient from non-consanguineous German parents (subject II-2; for pedigree, see Figure 1A; for clinical details see Table 1) presented with prelingual hearing loss since birth, recurrent severe infections from age 6 to 8 years with subsequent partially remitting bulbar paresis and flaccid tetraparesis which never fully resolved, and progressive visual loss due to optic atrophy since age 12 years (current vision at age 36 years: 0.1 right eye, 0.3 left eye). Gait and trunk ataxia started at age 30, leading to a dependency on a walking-frame at age 34 years. Disease severity on the Scale for the Assessment and Rating of Ataxia (SARA) yielded 14 out of 40 points [14]. Behavioural and mental disturbances started at age 16 years, yet the patient was still able to finish secondary school. Already at this time, the patient showed a severely reduced attention span, mental rigidity, low frustration tolerance and aggression to persons including family members. He was unable to perceive and describe his own physical problems during medical visits, rejected social contacts, and neglected the need for medical and rehabilitative treatment. In the following years, he developed attacks of verbal and physical aggression, e.g. repeatedly pulling his mother down to the floor by her hairs at age 35 years. Cycles of aggressive and impulsive behaviour alternated rapidly with cycles of infantile-regressive behaviour, e.g. shouting and crying at age 35 years when confronted with the need for a medical blood sampling. The lack of compliance and cooperative behaviour led to the cessation of examinations required for pre-surgery evaluation for cochlea implants. His cognitive capacities and insight at age 36 years seemed to be below the stage of an adolescent, but no formal neuropsychological testing was possible given his severe hearing and visual impairments and lack of cooperative behaviour. The combined load of physical, mental and behavioural disturbances required an institutionalization in long-term care since age 35 years. Cerebral MRI demonstrated mild cerebellar and parietal cortical atrophy (Figure 2A,B). Nerve conduction studies showed florid predominant axonal sensorimotor neuropathy (Table 1) with positive sharp waves on EMG of the tibial anterior muscle. Repeated lab testing revealed markedly increased serum creatinkinase (>1000 U/l, ref < 190 U/l), probably caused by progressive muscle cell turnover due to florid peripheral neuropathy. In addition, liver transaminases were constantly mildly elevated (AST 60 U/l; ref <50 U/l; ALT 90 U/l; ref < 50 U/l), with abdominal sonography showing no structural liver abnormalities.

**Table 1:** **Clinical findings and further investigations in *PRPS1 *** **Gln277Pro mutation carriers**

**Subject**	**II.2**	**II.1**	**I.2**
Gender	m	f	f
Phenotypic cluster	CMTX5/Arts	DFN2	None
Current age	36	42	66
Age of onset first symptom	Congenital, hearing loss	Congenital, hearing loss	None
**Neurological**			
Mental retardation	(+) progressive aggressive, childish behavior, starting at age 18	-	-
Ataxia (age of onset)	+, 30 years	-	-
Severity of ataxia (SARA score)	14 out of 40	1 out of 40	0 out of 40
Delayed motor development	-	-	-
Loss of deep tendon reflexes	+	-	-
Hearing loss (age of onset)	+, congenital	+, congenital	
Optic atrophy (age of onset)	12	--	-
**Uric acid overproduction**			
Gout	-	-	-
Kidney stones	-	-	-
Renal failure	-	-	-
Serum uric acid (ref 3.4-7.0 mg/dl)	4.3 mg/dl	4.4 mg/dl	4.2 mg/dl
**Hematopoetic**			
Recurrent infections	(+), only from age 6 to 8 years	-	-
Anemia	-	-	-
**Other**			
Short stature	-	-	-
Self-injury	-	-	-
Early death	-	-	-
**Lab, electrophysiology and MRI investigations**			
Nerve conduction studies			
-Sensory nerve conduction	Sur: no SNAP; Rad: no SNAP	Sur: normal, Rad: normal	n.d.
Sural or radial nerve			
-Motor nerve conduction	Tib: borderline MNCV (40 m/s; ref > 40 m/s); Uln: reduced CMAP (2.1 mV; ref: > 4 mV); reduced MNCV (36 m/s; ref: > 50 m/s)	Tib: normal; Uln: normal	
Tibial or ulnar nerve			
MRI	Mild cerebellar and parietal cortical atrophy	Mild cerebellar and parietal cortical atrophy	n.d.
Serum creatinkinase (ref <180 U/l)	1168 U/l	141U/l	196 U/l
PRS-I enzyme activity (ref 0.41-1.46 nmol/(min.mg protein)	<0.005 nmol/(min.mg protein)	0.1 nmol/(min.mg protein)	1.04 nmol/(min.mg protein)

This table was designed to show not only those clinical symptoms that are present, but also those that are absent in this index family, yet common in other *PRPS1* patients. The features were selected and extended from de Brouwer et al., 2010 [6]. Legend: m, male; f, female; n.d., not done; ref, reference value; SARA, scale for the Assessment and Rating of Ataxia, reaching from 0 to 40, with higher scores indicating more severe ataxia; scores < 3 points are considered unspecific. Sur = sural; Rad = radial; Tib = tibial, Uln = ulnar; SNAP = sensory nerve action potential; SNCV = sensory nerve conduction velocity; CMAP = compound muscle action potential; MNCV = motor nerve conduction velocity; MRI, magnetic resonance imaging.

His sister (subject II-1; Figure 1A) had developed prelingual sensorineural hearing loss in the first years of life, without any further signs of neurological dysfunction at current age of 42 years. This phenotype is fully compatible with the diagnosis of X-linked non-syndromic sensorineural hearing loss (DFN2) [5]. Her MRI showed discrete parietal and cerebellar atrophy as well (Figure 2C,D). The mother of the two subjects (subject I-1) did not show any hearing deficit or neurological dysfunction at the current age of 66 years. Uric acid in serum was normal in all three subjects (for an overview of all clinical and lab details, see Table 1).

### Serum biomarker and genetic analyses

Based on the initial clinical assumption of an autosomal-recessive ataxia, the index patient was assessed by extensive serum testing for biomarkers indicative of recessive ataxias (alpha-feto protein, lactate, vitamin E, very long chain fatty acids, phytanic acid, coeruloplasmin, cholesteanol, lysosomal enzymes, carbohydrate-deficient transferrin, quantitative assessment of amino acids) [15], and genetic testing of *FRDA*, *POLG*, *PEO1/Twinkle*, *WFS1*, and *ABHD12*. All results were normal. Sequencing of *PRPS1* revealed a novel hemizygous c.830A > C, p.Gln277Pro variant changing a highly conserved amino acid residue (PhyloP score 4.6; Figure 1C), predicted to be damaging by all *in silico* predictions (PolyPhen-2 score: 0.991; SIFT score: 0.01; MutationTaster: disease causing), and not found in 4,200 ethnically-matched control X-chromosomes or in the 6,500 exomes (= 10,562 alleles) of the NHLBI Exome Variant Server (EVS). The same variant was identified in the sister (II-1) and the mother (I-1) of the index patient in a heterozygous state (for electropherograms, see Additional file 3).

### Protein structural effects of the PRPS1 mutation

Gln277 is located in the C-terminal domain of *PRPS1* and is close to the catalytic active site, which consists of an ATP binding site and a ribose-5-P binding site (Figure 1D). The p.Gln277Pro change results in two sequential prolines, which very likely disrupts the alpha-helical structure of this short helical segment and destabilizes the C-terminal *PRPS1* domain. In addition, Gln277 interacts with Gly251, which is close proximity to the R5P binding site (Figure 1E). The p.Gln277Pro change will result in a loss of this hydrogen bonding interaction potentially destabilizing the region surrounding the ribose-5-P binding site, thus affecting the *PRPS1* catalytic active site. When comparing the protein structural effects of all known *PRPS1* mutations and their relations to *PRPS1*-phenotypic clusters, the p.Gln277Pro (Q277P) mutation falls in between CMTX5 and Arts syndrome *PRPS1* mutations. Like other Arts syndrome mutations (and in contrast to CMTX5 *PRPS1* mutations), it is predicted to disrupt the local *PRS-I* structure. However, unlike the other Arts syndrome mutations and in line with the CMTX *PRPS1* mutations, it is not predicted to disturb the allosteric site II or to affect the dimer interface (Table 2).

**Table 2:** **Predicted structural effects of known *PRPS1 *** **mutations**

**Mutation**	**Disturbance of local structure**	**Affecting dimer interface**	**Affecting trimer interface**	**Disturbance of ATP binding site**	**Disturbance of allosteric site I**	**Disturbance of allosteric site II**	**Neuropathy**
**PRS-I superactivity**							
p.D52H	+	-	+	-	+	-	-
p.N114S	-	+	+	-	+/-	+/-	+
p.L129I	+	-	-	-	-	+	+
p.D183H	-	+	-	-	+/-	+/-	+
p.A190V	+	+	-	-	+/-	+/-	+
p.H193L**	-	+	-	-	+/-	+/-	-
p.H193Q	-	+	-	-	+/-	+/-	+
p.V142L	+	+	-	-	-	+	+
**Arts syndrome**							
p.Q133P	+	+	-	+	+/-	+	+
p.L152P	+	+	-	+	+/-	+	+
**p.Q277P**	**+**	**-**	**+/-**	**+**	**-**	**-**	**+**
**CMTX5**							
p.E43D	-	-	+	+	+	-	+
p.M115T	-	-	+	+	+	-	+
p.A121G	+	-	+/-	+/-	+/-	-	+
**DFN2**							
p.D65N	**-**	**-**	**+**	**-**	-	-	?
p.A87T	**+**	**-**	**-**	**-**	-	-	?
p.I290T	**+**	**-**	**-**	**-**	-	-	?
p.G306R	**-**	**-**	**+**	**-**	-	-	?

When comparing the protein structural effects of all known PRPS1 mutations and their relations to PRPS1-phenotypic clusters, the p.Gln277Pro mutation falls in between CMTX5 and Arts syndrome *PRPS1* mutations. Like other Arts syndrome mutations (and in contrast to CMTX5 *PRPS1* mutations), it is predicted to disrupt the local PRS-I structure. However, unlike the other Arts syndrome mutations (and in line with other CMTX *PRPS1* mutations), it is not predicted to disturb the allosteric site II or to affect the dimer interface. This table was selected, updated and extended from de Brouwer et al., 2010 [6]. Please see here also for further references [6]. **This mutation was found in a female patient.

### Enzyme activity

PRS-I activity was not detectable in erythrocytes the index patient II-2 (<0.005 nmol/[min.mg protein]; reference value: 0.41-1.46 nmol/[min.mg protein]), reduced to 0.10 nmol/[min.mg protein] in his sister (II-1), and normal in their mother (I-1; 1.04 nmol/[min.mg protein]) (Figure 1B).

### X- chromosome inactivation (XCI)

XCI was extremely skewed (defined as ratio > 90%:10% [16]) in II-1 (94%:6%), but only moderately skewed in I-1 (80%:20%).

## Discussion

Here we report a family with a novel *PRPS1* mutation which provides new insights in *PRPS1*-related disease. The index patient (subject II-2) showed an overlapping phenotype combining features of both CMTX5 and (mild, relatively late-onset) Arts syndrome. Prelingual hearing loss, optic atrophy with onset in the teens and severe sensorimotor neuropathy are part of the classical CMTX5 trias [2]. However, recurrent infections in childhood leading to acute deteriorations of slowly progressive muscle weakness, severe ataxia leading to walker-dependency, mild to moderate mental and behavioural deficits, and institutionalization in homes for physically and mentally handicapped are all features not reported in previous CMTX5 subjects, but known from subjects with Arts syndrome (though usually in a more severe early-onset form) [3,17]. This shows that CMTX5 and Arts syndrome can overlap within one and the same individual, thus indicating a continuous spectrum of PRS1- hypoactivity disease. This finding corroborates and extends the recent observation from one individual with PRS-I superactivity who showed not only signs of PRS-I superactivity syndrome, but also of Arts syndrome [18]. Taken together, these findings indicate that both PRS1 superactivity and PRS-I hypoactivity disorders form a continuous gradual spectrum of disease.

The notion of a continuous disease spectrum of PRS1-hypoactivity disorders is further supported by our observation that a CMTX5/Arts overlapping phenotype can co-occur within the same family as a DFN2 phenotype (subject II-1), demonstrating an intrafamilial continuum of these three clusters of PRS-I hypoactivity diseases. So far, CMTX5, Arts syndrome and DFN2 were only reported in different families, not within the same family [6]. An earlier report already indicated that some subjects can present with an only incomplete presentation of a PRPS1 disease cluster (e.g. absence of optic atrophy in CMTX5 subjects [7]). Taken together, these findings suggest a continuous spectrum of *PRPS1* disease features, where hearing loss, peripheral neuropathy, optic atrophy, ataxia, cognitive deficits and recurrent infections are central, yet variable phenotypic features. Along this spectrum, the established *PRPS1* disease clusters are not separate entities, but endophenotypes on a phenotypic continuum.

Female carriers in families with Arts syndrome were known to sometimes exhibit some hearing impairment later in life (age >20 years) combined with ataxia and neuropathy [3,19]; yet prelingual hearing loss starting at birth - as observed in subject II-1 - has not yet been described in these families. Female carriers in families with CMTX5 are noted to be asymptomatic [1]. Our findings indicate that these current notions and corresponding counselling recommendations of female carriers in families with CMTX5 [1] or Arts syndrome [3] should be revised: female carriers in families with these phenotypic clusters can show symptoms already very early in life, leading to substantial impairment in everyday social life.

How might this intrafamilial continuum of *PRPS1* disease clusters be explained? Our results indicate that the respective phenotypic presentation seems to be determined by (1.) the exact *PRPS1* mutation (in males) and (2.) the residual enzyme activity, which in turn is largely influenced by the degree of skewed X-inactivation (in females). The predicted structural effects of the p.Gln277Pro mutation fall in between those of the CMTX5 and Arts syndrome *PRPS1* mutations (Table 2), thus explaining the CMTX/Arts syndrome overlapping phenotype in the male index patient II-2. PRS-I enzyme activity was not detectable in this male subject with the most complex and severe phenotype, while it was moderately reduced in his sister II-1 with a nonsyndromic hearing loss phenotype (DFN2), and normal in his unaffected mother I-1. (Figure 1B). The lack of detectable PRS-I activity in subject II-2 might explain why his phenotypic presentation included components of the Arts cluster, as absence of PRS-I activity has already been reported for patients with a pure Arts phenotype [4]. The residual PRS-I activity in the female carriers (I-1; II-1) might be explained by compensation through the second (intact) X chromosome. PRS-I activity was lower in subject II-1 than in subject I-1, most likely due to severely skewed X-chromosome inactivation which was observed in this subject. Alternatively, tissue-specific control of other genetic and epigenetic mechanisms might explain the differences in PRS1 activity between I-1 and II-1, e.g. the regulation of *PRPS1* by microRNA-376 [17] and difference in expression levels and function of the other three PRS isoforms [6]. Our finding of a clinico-enzymatic correlation between enzymatic activity and disease severity indicates that PRS-I activity may serve as a blood biomarker for *PRPS1* disease and may help to corroborate pathogenicity of novel *PRPS1* variants of unknown significance. It also suggests that it might serve as an interesting readout and surrogate parameter for treatment efficacy.

So far, cerebral MRI abnormalities have been observed neither in CMTX5 nor in Arts syndrome [3], yet such abnormalities are likely given the wide range of clinical central nervous abnormalities in *PRPS1*-disorders, in particular in Arts syndrome. Here we show that mild signs of cerebellar and parietal atrophy can be seen in both subject II-2 and subject II-1, thus providing first evidence for structural central nervous system damage in *PRPS1*- disease. As atrophy is very mild, it might have been present also in previous *PRPS1* subjects, yet not been recognized. Our finding of a subject with adolescent-onset ataxia with neuropathy, optic atrophy, hearing loss, and mild atrophy of the cerebellum without evidence of neurological disease in the parental generation places *PRPS1*-disease in the differential diagnosis of many adult-onset autosomal-recessive and mitochondrial ataxias where such findings are common and which might be mimicked by *PRPS1*-disease. This differential diagnosis includes e.g. OPA3/Type III methylglutaconic aciduria (MIM 606580), OPA1 (MIM 605290) or Wolfram Syndrome (MIM 606201).

## Conclusions

Our findings indicate that CMTX5, Arts syndrome and DFN2 are phenotypic clusters on a continuous intraindividual and intrafamilial spectrum of PRPS1-disease. The respective phenotypic presentation seems to depend on the exact *PRPS1* mutation and the residual enzyme activity and skewed X-inactivation, respectively.

## Abbreviations

CMTX5: Charcot-Marie-Tooth disease 5; DFN2: X-linked non-syndromic sensorineural deafness; EMG: Electromyography; MRI: Magnetic resonance imaging; PRS1: Phosphoribosylpyrophosphate synthetase 1.

## Competing interests

Dr. Synofzik received honoraria from Actelion Pharmaceuticals Ltd.

Dr. J. Müller vom Hagen received honoraria from Actelion Pharmaceuticals Ltd.

Dr. Haack reports no disclosures.

Dr. Wilhelm reports no disclosures.

Dr. Lindig received a travel grant by Bayer HealthCare.

Dr. Beck-Wödl received a travel grant by Actelion Pharmaceuticals Ltd.

Dr. Nabuurs reports no disclosures

Dr. van Kuilenburg reports no disclosures.

Dr. de Brouwer reports no disclosures.

Dr. Schöls reports no disclosures.

## Authors’ contributions

MS: conceptualization of the study, acquisition of data, analysis of the data, drafting the manuscript. JMvH: acquisition of data, revising the manuscript. TBH: acquisition of data, revising the manuscript. CW: acquisition of data, revising the manuscript. TL: acquisition of data, revising the manuscript. SB-W: acquisition of data, revising the manuscript. SBN: acquisition of data, revising the manuscript. ABPvK: acquisition of data, revising the manuscript. APMdB: acquisition of data, revising the manuscript. LS: supervision of the study, acquisition of data, revising the manuscript. All authors read and approved the final manuscript.

## Supplementary Material

Additional file 1 PRS-I Activity Analysis. Click here for file

Additional file 2 PRPS1 sequencing by PCR (Sanger sequencing). Click here for file

Additional file 3 Electropherogramms of PRPS1 sequencing. Click here for file

## References

[B1] Kim JW Kim HJ Pagon RA, Adam MP, Bird TD Charcot-Marie-Tooth Neuropathy X Type 5 GeneReviews 1993/2011 Seattle (WA): University of Washington, Seattle Accessed on November 2nd, 2013

[B2] Kim HJ Sohn KM Shy ME Mutations in PRPS1, which encodes the phosphoribosyl pyrophosphate synthetase enzyme critical for nucleotide biosynthesis, cause hereditary peripheral neuropathy with hearing loss and optic neuropathy (cmtx5) Am J Hum Genet 2007 81 3 552 558 10.1086/519529 17701900 PMC1950833

[B3] de Brouwer APM Duley JA Christodoulou J Pagon RA, Adam MP, Bird TD Arts syndrome GeneReviews 1993/2011 Seattle (WA): University of Washington, Seattle Accessed on November 2nd, 2013

[B4] de Brouwer AP Williams KL Duley JA Arts syndrome is caused by loss-of-function mutations in PRPS1 Am J Hum Genet 2007 81 3 507 518 10.1086/520706 17701896 PMC1950830

[B5] Liu X Han D Li J Loss-of-function mutations in the PRPS1 gene cause a type of nonsyndromic X-linked sensorineural deafness, DFN2 Am J Hum Genet 2010 86 1 65 71 10.1016/j.ajhg.2009.11.015 20021999 PMC2801751

[B6] de Brouwer AP van Bokhoven H Nabuurs SB PRPS1 mutations: four distinct syndromes and potential treatment Am J Hum Genet 2010 86 4 506 518 10.1016/j.ajhg.2010.02.024 20380929 PMC2850427

[B7] Park J Hyun YS Kim YJ Exome sequencing reveals a novel PRPS1 mutation in a family with CMTX5 without optic atrophy J Clin Neurol 2013 9 4 283 288 10.3988/jcn.2013.9.4.283 24285972 PMC3840141

[B8] Zimprich A Benet-Pages A Struhal W A mutation in VPS35, encoding a subunit of the retromer complex, causes late-onset Parkinson disease Am J Hum Genet 2011 89 1 168 175 10.1016/j.ajhg.2011.06.008 21763483 PMC3135812

[B9] Adzhubei IA Schmidt S Peshkin L A method and server for predicting damaging missense mutations Nat Methods 2010 7 4 248 249 10.1038/nmeth0410-248 20354512 PMC2855889

[B10] Kumar P Henikoff S Ng PC Predicting the effects of coding non-synonymous variants on protein function using the SIFT algorithm Nat Protoc 2009 4 7 1073 1081 19561590 10.1038/nprot.2009.86

[B11] Schwarz JM Rodelsperger C Schuelke M MutationTaster evaluates disease-causing potential of sequence alterations Nat Methods 2010 7 8 575 576 10.1038/nmeth0810-575 20676075

[B12] Li S Lu Y Peng B Crystal structure of human phosphoribosylpyrophosphate synthetase 1 reveals a novel allosteric site Biochem J 2007 401 1 39 47 10.1042/BJ20061066 16939420 PMC1698673

[B13] Allen RC Zoghbi HY Moseley AB Methylation of HpaII and HhaI sites near the polymorphic CAG repeat in the human androgen-receptor gene correlates with X chromosome inactivation Am J Hum Genet 1992 51 6 1229 1239 1281384 PMC1682906

[B14] Schmitz-Hubsch T du Montcel ST Baliko L Scale for the assessment and rating of ataxia: development of a new clinical scale Neurology 2006 66 11 1717 1720 10.1212/01.wnl.0000219042.60538.92 16769946

[B15] Anheim M Tranchant C Koenig M The autosomal recessive cerebellar ataxias N Engl J Med 2012 366 7 636 646 10.1056/NEJMra1006610 22335741

[B16] Sharp A Robinson D Jacobs P Age- and tissue-specific variation of X chromosome inactivation ratios in normal women Hum Genet 2000 107 4 343 349 10.1007/s004390000382 11129333

[B17] Liu XZ Xie D Yuan HJ Hearing loss and PRPS1 mutations: wide spectrum of phenotypes and potential therapy Int J Audiol 2013 52 1 23 28 10.3109/14992027.2012.736032 23190330 PMC4511087

[B18] Moran R Kuilenburg AB Duley J Phosphoribosylpyrophosphate synthetase superactivity and recurrent infections is caused by a p.Val142Leu mutation in PRS-I Am J Med Genet A 2012 158A 2 455 460 10.1002/ajmg.a.34428 22246954

[B19] Arts WF Loonen MC Sengers RC X-linked ataxia, weakness, deafness, and loss of vision in early childhood with a fatal course Ann Neurol 1993 33 5 535 539 10.1002/ana.410330519 8498830

