# Supplementary material for: X-linked Charcot-Marie-Tooth disease, Arts syndrome, and prelingual non-syndromic deafness form a disease continuum: evidence from a family with a novel PRPS1 mutation
Source: Orphanet J Rare Dis. 2014 Feb 14;9:24. doi: 10.1186/1750-1172-9-24 (PMC3931488; doi:10.1186/1750-1172-9-24)
Supplement: Additional file 1 — PRS-I Activity Analysis. [file 1750-1172-9-24-S1.doc]

**Supplement 2: PRS-I Activity Analysis**

One hundred microliters of frozen-packed erythrocytes was diluted with 400 µl 0.9% (w/v) NaCl and concentrated on an Amicon Ultra Ultracel 10K Membrane filter (Millipore) by centrifugation (14,000g at 4°C for 60 min). The protein fraction was diluted to a final volume of 500 µl with 0.9% (w/v) NaCl and concentrated again by centrifugation. This procedure was repeated once more and the protein concentration determined with a copper-reduction method using bicinchonic acid.

The activity of PRS-I was determined in a reaction mixture (110 µl) containing an aliquot of cell sample, 32 mM sodium phosphate, 1 mM dithiothreitol, 4.5 mM MgCl_2_, 1 mM ATP, 100 µM ribose-5-phosphate and 50 mM TRIS/MOPS (pH7.4). Separation of AMP, ADP and ATP was performed using a gradient from 100 % buffer A [7.5 mM sodium phosphate] to 70 % buffer B [0.75 M sodium phosphate (pH 4.55)] in 25 min, at a flow rate of 1.0 ml/min by HPLC on an ion exchange column (Whatman Partisphere SAX 125 x 4.6 mm, 5 µm particle size) and a guard column (Whatman Partisphere AX 10 x 2.5 mm, 5 µm particle size) with online UV detection at 254 nm.
