# Supplementary material for: X-linked Charcot-Marie-Tooth disease, Arts syndrome, and prelingual non-syndromic deafness form a disease continuum: evidence from a family with a novel PRPS1 mutation
Source: Orphanet J Rare Dis. 2014 Feb 14;9:24. doi: 10.1186/1750-1172-9-24 (PMC3931488; doi:10.1186/1750-1172-9-24)
Supplement: Additional file 2 — PRPS1 sequencing by PCR (Sanger sequencing). [file 1750-1172-9-24-S2.doc]

**Supplement 1: *PRPS1* sequencing by PCR (Sanger sequencing)**

We performed a mutation screening of the genomic DNA of all coding exons in the *PRPS1* gene (NM_002764.3), including flanking intron sequences by polymerase chain reaction (AmpliTaq Gold® Fast PCR Master Mix, Invitrogen, Darmstadt, Germany), using the primers given below. After initial denaturation at 95°C for 5 min, each cycle consisted of denaturation at 95°C for 15 sec, primer annealing at 64°C for 15 sec and extension at 72°C for 40 sec. Sequencing was carried out using the BigDye® Terminator v3.1 Cycle Sequencing Kit on ABI 3730 DNA Analyzer as recommended by the user guide (Applied Biosystems).

Amplification and sequencing of the PRPS1 exons using the following primer pairs:

PRPS1-Ex01F;GCGCCGGGCGGGAATGTAAG

PRPS1-Ex01R;GTCACAGAGCTGCACCCTCTCCCC

PRPS1-Ex02F;GCGCCCAGCCCTGCCATATACTTTA

PRPS1-Ex02R;TCCAGTACTCCAGAGGAGTTGGTGCTTAG

PRPS1-Ex03F;TGGACATGTCTCCTTCTATGAATTTCTGGG

PRPS1-Ex03R;AACTAGACTGCCTCCCTATCTAACCACCTG

PRPS1-Ex04F;CACTGGGCCTGCCTTCCCATC

PRPS1-Ex04R;CAACCCATGTGCTAGCTACTTACATCCATTC

PRPS1-Ex05F;TTCTCCTCCCCAAAACAAGCCCATT

PRPS1-Ex05R;TCTCACAAAGTTACTTATCCCCTCAATTTGGTTC

PRPS1-Ex06F;GGCCTCAGCATGACACCTACTTTGTCTG

PRPS1-Ex06R;ACAAATTCTGATGACAAGACTAAATCCTTCAGACC

PRPS1-Ex07F;CAGCCTCATGACAGGGAAACAGCA

PRPS1-Ex07R;TCATCAAATACCAAGGGGAAACAAGGGTG
