# Supplementary material for: X-linked Charcot-Marie-Tooth disease, Arts syndrome, and prelingual non-syndromic deafness form a disease continuum: evidence from a family with a novel PRPS1 mutation
Source: Orphanet J Rare Dis. 2014 Feb 14;9:24. doi: 10.1186/1750-1172-9-24 (PMC3931488; doi:10.1186/1750-1172-9-24)
Supplement: Additional file 3 — Electropherogramms of PRPS1 sequencing. [file 1750-1172-9-24-S3.doc]

**Supplement 3: Electropherogramms of *PRPS1* sequencing**


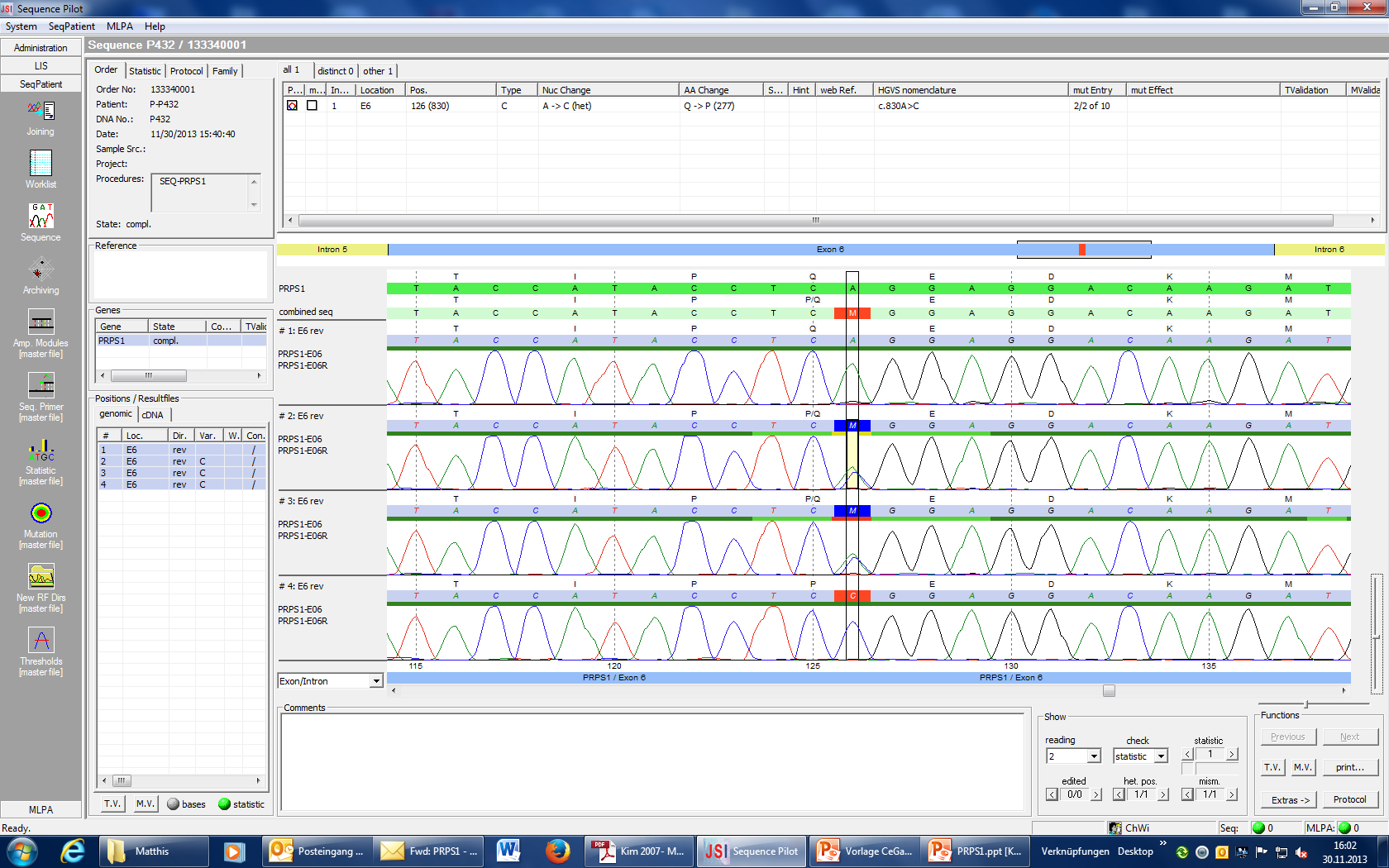


**control**

**sister (II.1)**

**mother (II.2)**

**index patient (II.2)**

The chromatograms of the c.830A>C; p.Gln277Pro variant in exon 6 of the *PRPS1* gene detected by sequencing in a male index patient (II.2). His sister (II.1) and mother (I.2) are heterozygous carrier of the mutation.
